# Sources, fate and distribution of inorganic contaminants in the Svalbard area, representative of a typical Arctic critical environment–a review

**DOI:** 10.1007/s10661-021-09305-6

**Published:** 2021-10-14

**Authors:** Paulina Rudnicka-Kępa, Agata Zaborska

**Affiliations:** Institute of Oceanology Polish Academy of Sciences, Sopot, Poland

**Keywords:** Heavy metals, Contaminants, Pollution, Arctic, Polar regions

## Abstract

Global environmental changes not only contribute to the modification of global pollution transport pathways but can also alter contaminant fate within the Arctic. Recent reports underline the importance of secondary sources of pollution, e.g. melting glaciers, thawing permafrost or increased riverine run-off. This article reviews reports on the European Arctic–we concentrate on the Svalbard region–and environmental contamination by inorganic pollutants (heavy metals and artificial radionuclides), including their transport pathways, their fate in the Arctic environment and the concentrations of individual elements in the ecosystem. This review presents in detail the secondary contaminant sources and tries to identify knowledge gaps, as well as indicate needs for further research. Concentrations of heavy metals and radionuclides in Svalbard have been studied, in various environmental elements since the beginning of the twentieth century. In the last 5 years, the highest concentrations of Cd (13 mg kg^−1^) and As (28 mg kg^−1^) were recorded for organic-rich soils, while levels of Pb (99 mg kg^−1^), Hg (1 mg kg^−1^), Zn (496 mg kg^−1^) and Cu (688 mg kg^−1^) were recorded for marine sediments. Increased heavy metal concentrations were also recorded in some flora and fauna species. For radionuclides in the last 5 years, the highest concentrations of ^137^Cs (4500 Bq kg^−1^), ^238^Pu (2 Bq kg^−1^) and ^239 + 240^Pu (43 Bq kg^−1^) were recorded for cryoconites, and the highest concentration of ^241^Am (570 Bq kg^−1^) was recorded in surface sediments. However, no contamination of flora and fauna with radionuclides was observed.

**Supplementary information:**

The online version contains supplementary material available at 10.1007/s10661-021-09305-6.

## Introduction

Despite the low urbanization of the Arctic, an important amount of pollutants gets into this region, from sources that can be even thousands of kilometres away (Macdonald et al., 2005). By the 1960s, contaminants had already been found in the tissues of Arctic marine mammals (Holden & Marsden, 1967). A breakthrough was a finding of organic contaminants (polychlorinated biphenyls—PCBs) in Canadian Inuit mothers’ milk and information about the large amount of radionuclides dumped in the Kara Sea by the former USSR (Macdonald, 2001). In 1991, the Arctic Monitoring and Assessment Program (AMAP) was created, to identify pollution and assess its impact on Arctic ecosystems. The result was the first report published in 1997 (AMAP, 1997), which demonstrated a close connection between the Arctic and the rest of the world, caused by the transport of pollutants over long distances. Subsequent research led to further assessment of contamination of the Arctic environment (e.g. AMAP (2002, 2004, 2005, 2006, 2010); Braune et al. (1999); Muir et al. (1999); van Oostdam et al. (1999); Macdonald et al., (2000, 2005, 2010) and, more recently, AMAP (2011, 2016, 2019) and Fong-McMaster et al. (2020)). A recent report on pollution and its sources, prepared by the Norwegian Polar Institute to identify corrective actions and evaluate current methods, pointed out the widespread problem of long-range pollution in Svalbard and indicated the lack of a system enabling the classification and risk assessment for the Arctic (Granberg et al., 2017).

The first reports confirming secondary pollution of the environment, along with the melting of glaciers, appeared in 2001, when Blais et al. (2001) confirmed the release of persistent chloroorganic compounds from a glacier into Lake Alberta, in Canada. It has also been suggested that, as the climate warms, pollutants previously deposited on glacier surfaces will be released. This phenomenon may be even more important in the future now that a twofold increase in temperature, compared to the global average over the last two decades, has been found in the Arctic (Richter-Menge et al., 2019). During the winter months in 2016 and 2018, it was observed that the surface temperature in the central Arctic was 6 °C higher than the average from 1981–2010 (Overland et al., 2018), and in the years 2006–2015, there was a loss of glaciers in the Arctic at a rate of − 213 ± 29 Gt year^−1^, of which the figure at Svalbard and Jan Mayen Island was − 9 ± 5 Gt year^−1^ (Meredith et al., 2019). Arctic glaciers can store pollution, even that from antiquity (Hong et al., 1994), and thus re-introduction of those pollutants to the environment can become an emerging issue.

This review article concentrates on the European Arctic (Svalbard) and reports on environmental contamination by inorganic pollutants (heavy metals and artificial radionuclides), including their transport pathways, their fate in the Arctic environment and the concentrations of individual elements in the ecosystem. This article presents in detail the secondary contaminant sources, tries to identify knowledge gaps and also indicates needs for further research.

### Svalbard–a flagship point in the Arctic

The Svalbard area is believed to be a representative of a typical Arctic critical environment (Dallmann, 2015). Due to the location of its archipelago and easy access, Svalbard is a key place for observing the Arctic environment in general, including the impact of climate change, glaciology, geology or biodiversity. Research in Svalbard is characterized by a high degree of international cooperation (the international research station in Ny-Ålesund, UNIS, SIOS) and is an important flagship site for the European Environment Agency and numerous scientific institutions.

The Svalbard archipelago, situated in the European Arctic, is approximately 57% covered by glaciers (~ 34,000 km^2^), which corresponds to around 10% of the total area of glaciers in the entire Arctic. The total volume of ice in Svalbard has been estimated to be ~ 6200 km^3^ (Fürst et al., 2018). Around 60% of these glaciers (by surface) are tidal glaciers, ending in a fjord or the ocean (Schuler et al., 2020). This type of glacier introduces freshwater into the marine ecosystem through subglacial channels, as well as from submarine melting and the melting of icebergs calved off the glacier fronts. For 20 years, an increasingly fast loss of glacier mass has been observed (−8 ± 6 Gt year^−1^; Schuler et al., 2020). Those most sensitive to temperature changes are the thinner and smaller glaciers with a small area of accumulation zone (northern and central Svalbard). In turn, thicker and larger polythermal glaciers, which account for the majority in Svalbard (Nowak et al., 2021), can store, transport and release water even during the winter. Part of the non-glaciarized terrain on Svalbard consists of landforms such as alluvial fans, blockfields, expansive areas with raised marine sediments, colluvial fans covering steeper slopes and sets of beach ridges (Allaart et al., 2021). Intensively melting glaciers are accompanied by a thawing permafrost layer in non-glaciarized areas. In the years 2016–2019, a decrease in the active layer thickness was observed at most of the observation sites in Svalbard (up to 6.5 cm year^−1^ in Adventdalen; Moreno-Ibáñez et al., 2021). The primary sources of freshwater to fjords are melting glaciers and sea ice, local rainfall and river runoff. In Svalbard, a large number of rivers are fed by melting glaciers and thawing permafrost. River runoff on Svalbard is highly seasonal–the most significant runoff occurs between May and September–and most rivers freeze during the winter period. Considering that river flow in Svalbard depends on an increase in glacier melting, rainfall and the melting of permafrost, it makes rivers there particularly vulnerable to climate change (Moreno-Ibáñez et al., 2021). However, the latest analyses carried out by Nowak et al. (2021) indicate that the catchments with smaller, significantly retreated glaciers, have already reached so-called peak water and are located on the descending section of the runoff curve (e.g. Waldemarelva, Greerelva, Bayelva). On the other hand, catchments affected by larger glaciers–and possibly a more significant part of the non-glaciarized area–are still impacted by increased freshwater runoff (e.g. Adventelva).

Arctic fjords, such as those in Svalbard, are an interface between terrestrial and marine environments and are particularly vulnerable to climate change. These aquatic critical zones (Bianchi et al., 2020) are influenced by glaciers, surface waters and human activities which control the sources, transport and fate of contaminants (Zaborska, 2017; Zaborska et al., 2017).

The major contaminant groups in the Arctic are persistent organic pollutants (POPs), heavy metals and artificial radionuclides (AMAP, 1997). In this paper, we concentrate on inorganic pollutants: heavy metals and radionuclides. Natural sources of heavy metals in the environment include, for example, rock weathering, volcanic eruptions and forest fires. However, the highest emission of metals to the environment is caused by human activities. The most important anthropogenic sources include coal and waste combustion, heat and power plants, transport and the metallurgical and chemical industry (Liu et al., 2018). It has been proven that most of the metals present in the atmosphere over Svalbard are emitted in Europe and Russia (Ardini et al., 2020; Isaksen et al., 2016). The largest sources of anthropogenic radionuclides are global fallout from nuclear tests carried out in the 1950s and 1960s, nuclear fuel processing in Western Europe (Sellafield, Cape La Haque), the Chernobyl power plant disaster (1986) and nuclear installations and waste dumps in the Arctic (Novaya Zemlya) and within the catchment areas of the Ob and Yenisei rivers (Łokas et al., 2013). In addition, there is also some pollution of local origin, e.g. from coal mining (Khan et al., 2017), sewage discharge (Kalinowska et al., 2020) and transport (Zhan et al., 2014).

The problem of environmental contamination in Svalbard is becoming more important nowadays in a time of ongoing climate change. It is estimated that the climate on the west coast of Spitsbergen is warming more than six times faster than the global average (Wawrzyniak & Osuch, 2020). Global environmental changes not only contribute to changes in pollution transport pathways but also lead to an increase in the fluxes of pollutants entering the Spitsbergen aquatic environments from so-called secondary sources of pollution, e.g. melting glaciers, thawing permafrost or rivers (Kohler et al., 2007; Zaborska, 2017; Zaborska et al., 2017).

## Heavy metal and radionuclide transport pathways

Contaminants enter the Svalbard ecosystem from multiple sources. The most important are long-range pathways (atmospheric circulation, ocean currents, riverine transport and ice drift) and local pathways (e.g. coal mining). Moreover, releases from secondary sources (melting glaciers, thawing permafrost, riverine runoff and sea ice melting) lead to increased concentrations in aquatic environments.

### Long-range contamination pathways

#### Heavy metals

Of all the distant transport paths, air circulation is the fastest way to transfer contaminants to Svalbard from other regions of the world. Heavy metals of anthropogenic origin directly enter the atmosphere in the form of aerosols and particles. They are then transferred by atmospheric circulation to and over the Arctic and can then be released by dry deposition or precipitation, as well as during direct air/water/ice exchange (Durães et al., 2018). Heavy metal air concentrations regularly monitored on Svalbard by the Norwegian Institute for Air Research (NILU) have indicated smaller or larger decreases in the Arctic. Emissions of lead, mercury and cadmium decreased by 65%, 13% and 55% respectively from 1991 to 2019 at the Zeppelin Station (Spitsbergen) (Bohlin-Nizzetto et al., 2019).

Ocean currents are another pathway for transport of heavy metals directly into the Spitsbergen (Svalbard) marine environment, especially for dissolved heavy metals (e.g. Cd, Pb) (Gobeil et al., 2001; Maccali et al., 2013). It has been found that marine current transport of soluble contaminants from Europe to Svalbard takes about 5 years (Dahlgaard, 1995).

Heavy metals may also enter the Spitsbergen (Svalbard) via riverine runoff. The largest rivers discharging freshwater to the Arctic are the Yenisei, Lena and Ob. Rivers carry a substantial amount of pollutants leached out from industrial areas, e.g. the Siberian Chemical Combine. It is estimated that the average annual inflow from the nine largest rivers to the central Arctic Basin is around 2000 km^3^ year^−1^. Krickov et al. (2019) published the concentrations of trace elements in the suspended matter of 33 Siberian rivers. The metal concentrations found were very variable and exceeded the natural background concentrations: for example, Cd levels were 0.1–1.2 mg kg^−1^, levels of As were 1–95 mg kg^−1^ while Pb levels were 5–45 mg kg^−1^. Stein (2008) stated that up to 90% of heavy metals associated with particles transported along with river runoff settles in estuaries, though dissolved metal fractions are transported away from rivers.

The last major contaminant transport pathway is a drift of sea ice. This concerns material incorporated into sea ice during its formation as well as contaminants deposited directly on such ice from the atmosphere. Sea ice formed mainly at Siberian coasts is transported by the Transpolar Drift Stream, within 2–4 years, to the European Arctic (Pavlov et al., 2004; Su et al., 2019). Tovar-Sánchez et al. (2010) identified significant concentrations of heavy metals (especially Fe and Zn) in Arctic ice and suggested that ice melting is a significant source of pollutants. This theory has also been confirmed by the latest research conducted by Zaborska et al. (2020) on the sea ice in Hornsund (Spitsbergen, Svalbard).

#### Artificial radionuclides

The fastest way of transporting radionuclides, as with other pollutants, is through air circulation. Monitoring of radionuclides in the atmosphere is not so systematic, though accidental increases in radionuclide levels associated with accidents, such as in Chernobyl, or related to natural causes such as forest fires have been identified (AMAP 2016).

Long-range transport to Spitsbergen (Svalbard) by marine currents (the Norwegian Atlantic Current and the Norwegian Coastal Current) is the most important means for transport of soluble, conservative radionuclides (e.g. ^99^Tc, ^137^Cs) (Brown et al., 1999; Johannessen et al., 2010). The transport of radionuclides within the Arctic has been constantly studied and modelled by the Norwegian Radiation and Nuclear Safety Authority (DSA). Moreover, numerical models simulating transport in the marine environment are constantly being improved since the pioneering work of Prandle (1984), where the fate of ^137^Cs was simulated after being released from the Sellafield nuclear reprocessing plant (Periáñez et al., 2019).

Riverine concentrations of artificial radionuclides are subject to large fluctuations, depending on the current global situation: for example, the highest radionuclide levels were achieved after the explosion at the Chernobyl power plant (AMAP, 2004).

Multiyear sea ice has been found to transport radionuclides (e.g. Dethleff et al., 2000; Masque et al., 2007; Pavlov & Stanovoy, 2001). Masque et al. (2007) focused on the sources, fate and time scales of radionuclide transport by sea ice and suggested a need for more thorough research, as sea ice may be an important transport medium for contaminants, and also a secondary source. These studies were later continued by Cámara-Mor et al. (2010, 2011, 2012).

### Local sources

#### Heavy metals

Local sources of heavy metals in Svalbard are coal mines (Tolvanen et al., 2018), pollution from human activity, sewage (Hop et al., 2001; Kalinowska et al., 2020), leachate from landfills, coal combustion in power and heat plants (Khan et al., 2017; Rose et al., 2004) and harbours and coal transshipment docks (Van den Granberg et al., 2017; Heuvel-Greve et al., 2016). The last source includes transport and developing tourism is nowadays becoming an important local source (Eckhart et al., 2013; Zhan et al., 2014). Other sources of heavy metals in the Arctic, which can be delivered into Svalbard ecosystems along with long-range transport, are metal (Pb–Zn) mines in Greenland (Johansen et al., 1991; Elberling et al., 2002; Perner et al., 2010) and metal ore mining in northern Russia (Walker et al., 2003; Tkatcheva et al., 2004) and Europe (Heikkinen et al., 2002; Sternal et al., 2017).

#### Artificial radionuclides

Local sources of radionuclides in Svalbard were mining operations in the neighbourhood of Ny-Alesund (Dowdall et al., 2004). Currently, there are no sites in the territory of Svalbard that could directly introduce radionuclides in significant amounts into the environment. Other sources that may affect the Svalbard ecosystem are dumped radioactive materials and old submarines on Nowaya Zemlya island and the Kola peninsula (Baskaran et al., 1996; Smith et al., 2000), nuclear weapons tests and occasional radioactive isotope accidents (e.g. Chernobyl; Cwanek et al., 2020).

### Secondary sources–remobilization of contamination

The process of secondary contamination is associated with the re-release of contaminants previously deposited on the surface of the land, glaciers and/or surface waters. In this era of climate change, this process occurs with the melting of ice, glaciers and snow cover, thawing of permafrost, increased discharge of river runoff or extensive coastal erosion. Taking into consideration the increase in global temperature, and the related increasing rate of melting of glaciers and the increased amount of flowing river water, this phenomenon could have a significant impact on the hydrology and hydrodynamics of fjords, on sediment and contaminant flux and consequently on marine flora and fauna (Kim et al., 2020; Mohan et al., 2018; Zaborska et al., 2017, 2020). Research on secondary pollution of the Arctic environment has been carried out by, among others, Zdanowicz et al., (2013; Baffin Island (Canada)), Søndergaard et al., (2015; Northeast Greenland), Schuster et al., (2018; Alaska), Lim et al., (2020; West Siberia) and Perryman et al., (2020; Alaskan soils). In the case of Spitsbergen, this is a relatively new topic, and research related to secondary pollution sources in this area has only recently been conducted. The impact of environmental changes on the environment of Spitsbergen has been noticed, among others by Birks et al. (2004) for Spitsbergen lakes, Zaborska et al. (2017), Zaborska (2017) and Pouch et al. (2017, 2018, 2021) for marine sediments and Zaborska et al. (2020) for seawater. Mohan et al. (2018) investigated the effect of glacier melting waters on bottom sediments in Kongsfjorden while Johansen et al. (2021) concentrated on riverine suspended particulate matter (SPM) and marine sediments in Isfjorden. Isakson et al. (2003) examined ice cores collected at two stations in Svalbard (Lomonosovfonna and Austfonna) for historical contamination content (mainly organic). They pointed out the possible danger of pesticides being released into the aquatic environment along with melting ice and the dissociation of such compounds.

**Fig. 1:**
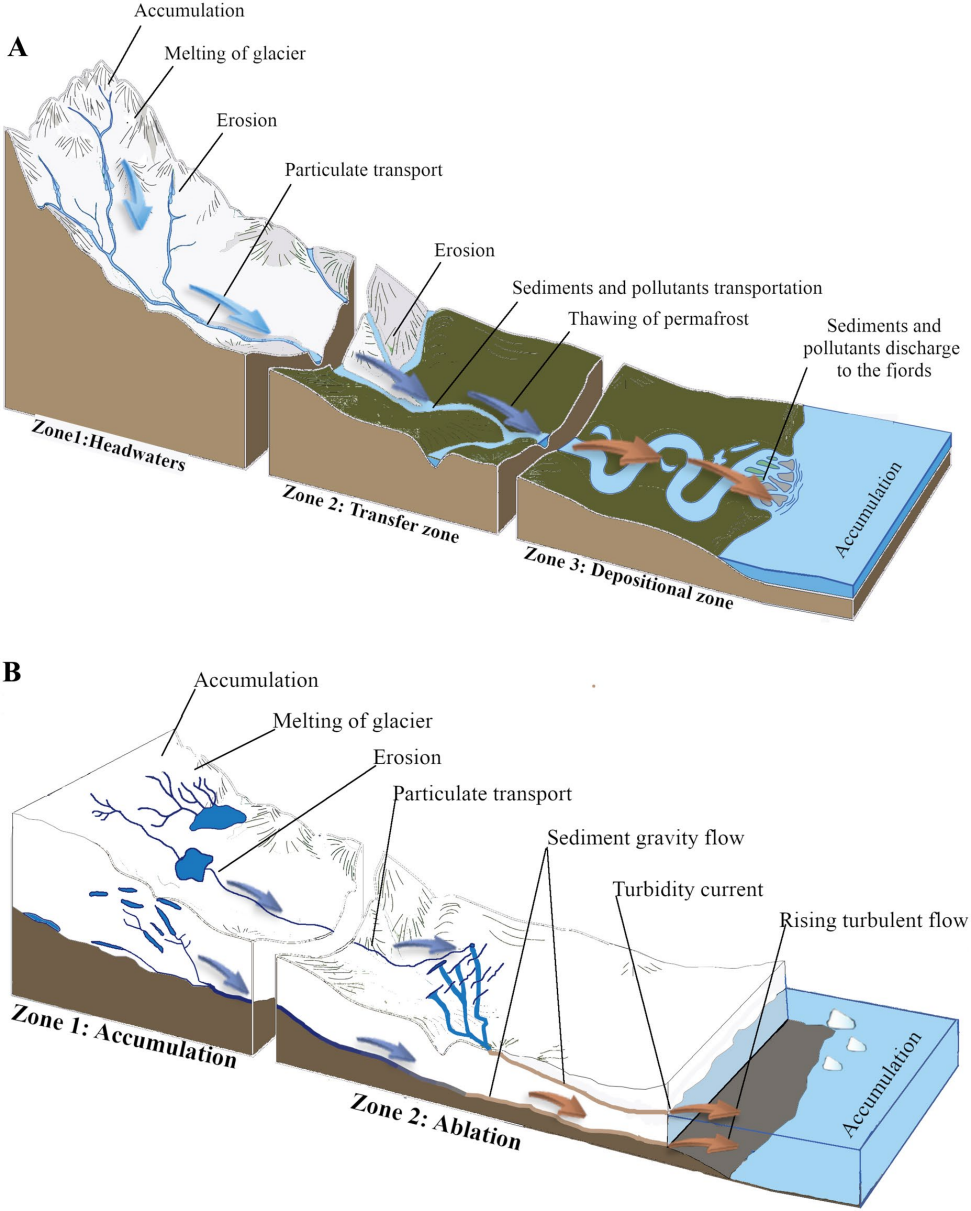
Secondary sources of contaminants to an Arctic fjord. **A** Melting land terminating valley glacier and thawing permafrost feed river. **B** Tidewater glacier delivers meltwater directly to fjord seawater by meltwater runoff and frontal ablation (based on Chu, 2014)

Several types of glacier are present on Svalbard: ice caps, land terminating valley glaciers and tidewater valley glaciers that flow down directly to the ocean (Fig. 1). Valley glaciers have two zones: the so-called accumulation zone, in which the mass of snow accumulates, and the second–the ablation zone–where the glacier loses its mass due to erosion, wind forces, precipitation, calving and melting (Chu, 2014). Airborne contaminants accumulate on the surface of glaciers via dry and wet deposition. In late spring and summer, water from accumulated snow and glacier ice melting channelizes into supraglacial streams and ponds (Fig. 1) (Chu, 2014). In the glacier ablation zone, meltwater drains through moulins, providing an influx of water into englacial and subglacial environments. In tidewater glaciers, subglacial meltwater conduits directly enter marine water at the level of the glacier base and through the underwater gates localized at the glacier front. Glacier meltwater is enriched in natural rock/soil material from bedrock erosion but also includes airborne material previously deposited at the glacier surface. Thus, contaminants of atmospheric origin accumulated at the glacier surface are transferred into the marine environment. Higher temperatures enhance the melting of not only seasonally formed snow cover but also of old glacier ice. Previously formed glacier ice can contain contaminants that have been accumulated at the glacier surface over the last century (Cogley et al., 2011; Spolaor et al., 2021). In a recent study, Mohan et al. (2018) showed the retreat of glaciers as a result of global warming and, with it, an increase in the runoff of meltwater containing high quantities of suspended solids in Kongsfjorden. They analysed the sedimentation rate in the inner fjord and it showed an increase over the last 20 years.

Research conducted by, for example, Węsławski et al. (1995), Beszczyńska-Möller et al. (1997), Hagen et al. (2003) and Błaszczyk et al. (2019), confirms that glaciers are the main source of freshwater supplied to the Spitsbergen fjords. The latest research by Błaszczyk et al. (2019) indicates that the average freshwater supply to Hornsund from tidewater glaciers is 257 ± 82 Mt year^−1^, with as much as 39% (986 Mt year^−1^) coming from glacier meltwater runoff and 25% (634 Mt year^−1^) from frontal ablation of tidewater glaciers. Snowfall in winter, rainfall and melting of snow cover represent 21%, 7% and 8% respectively, which seems to be a small contribution to the total freshwater load. Glacier ablation leads to enhanced pollution discharge from the glaciers to the marine environment (Cogley et al., 2011).

Fjord seawater receives enormous amounts of freshwater directly from melting glacier calving and underwater gates in the glacier front, as well as indirectly via river runoff. These rivers are fed by mountain glacier meltwater, snow melting, permafrost thawing and precipitation (St Pierre et al., 2018). It is estimated that, along with climate change, for every 2 °C increase in temperature, there will be a 22% increase in the sediment supply carried by rivers. For every 20% increase in water discharge in rivers, there will be a 10% increase in particulate load (Zajączkowski et al., 2004) which may lead to an increase in the flow of secondary pollutants in some regions of Svalbard (Kim et al., 2020). Very few studies have been carried out on the Svalbard rivers, though Bogen and Bonsnes (2003) focused on the erosion process and sediment transport along with the river runoff of Endalselva, Bayelva and Londonelva. An estimation of the variability of water/suspension (and potentially contaminant) discharge by Svalbard rivers would be very interesting, as Ye et al. (2009) and Song et al. (2019) indicated relationships between hydrological regimes and permafrost thawing in large Arctic rivers. The importance of sedimentary material discharge by a Svalbard river (Adventelva) was pointed out by Zajączkowski et al. (2004). McGovern et al. (2020) have recently shown, based on a study of Adventelva, that riverine discharge actually drives the distribution of substances within a fjord. A study by Kim et al. (2020) on total mercury concentrations transported with suspended solids to bottom sediments, in Spitsbergen fjords (Wijdefjorden, Dickonfjorden and Hornsund), suggests that rivers may increase the concentration of inorganic pollutants in the fjords’ marine ecosystem. Recent research by Johansen et al. (2021) on persistent organic pollutants in Isfjorden suggests, however, that the increased inflow of SPM from land may lead to the dilution of pollutants in coastal sediments while affecting their bioavailability in the marine food web.

Other minor processes leading to secondary contamination of Svalbard coasts include transport of metals of anthropogenic origin associated with marine aerosols (Kozak et al., 2015; Lüdke et al., 2005; Samecka-Cymerman et al., 2011). Marine air masses contain sea salt particles enriched in heavy metals due to fractionation in the sea-surface microlayer. It has been found that heavy metals originating from air mass transport prevail in the spring season while sea salt particle generation has higher importance in the summer and autumn (Shevchenko et al., 2003).

An interesting phenomenon concerns the transfer of pollutants by migratory bird species. This was first noticed at seabird colonies in Bjornøya by Evenset et al., (2004, 2007). Seabirds feed on marine species and then–through excretion–redistribute the contaminants to the terrestrial environment. This process was then confirmed by Kristiansen et al. (2019) who studied bird colonies in Kongsfjorden. Moreover, migratory species can transfer contaminants from highly contaminated wintering areas to the Arctic. Pacyna-Kuchta et al. (2020) and Albert et al. (2021) found that migratory birds travelling over long distances can transport toxic substances and via excretion introduce them into the Arctic. They indicated that the Hg concentrations in the bodies of birds in the non-breeding period were several times higher than those found in seabirds breeding in the West Atlantic. The Hg concentrations in the feathers of those birds reached a threshold at which adverse effects on the organism are observed.

Another pathway for secondary pollution of Svalbard shore waters is coastal erosion. Zagórski et al. (2015) found that coastal erosion is a significant process changing the coast of Isbjørnhamna (Hornsund, Svalbard). The coastal area during the observation (1960–2011) decreased by over 31,600 m^2^. An analysis by Wojtysiak et al. (2018) showed that the most intense storms occur in spring and autumn. Due to climate change, storms may occur more frequently and become stronger, and thus coastal erosion may become a more important secondary pollution source (Kim et al., 2020).

Groundwater discharge can also be an important source of pollution, as it has been shown to be an important source of nutrients, heavy metals and major ions to rivers and oceans (Webb et al., 2018). In the rapidly warming Arctic permafrost, thawing can intensify groundwater flow (Lecher, 2017; Nowak et al., 2021). In areas with contaminated soil and groundwater, increased groundwater flow may be significant for the functioning of the aquatic ecosystem (Evengard et al., 2011; Nowak et al., 2021).

## Fate of inorganic pollutants in the Arctic environment

**Fig. 2:**
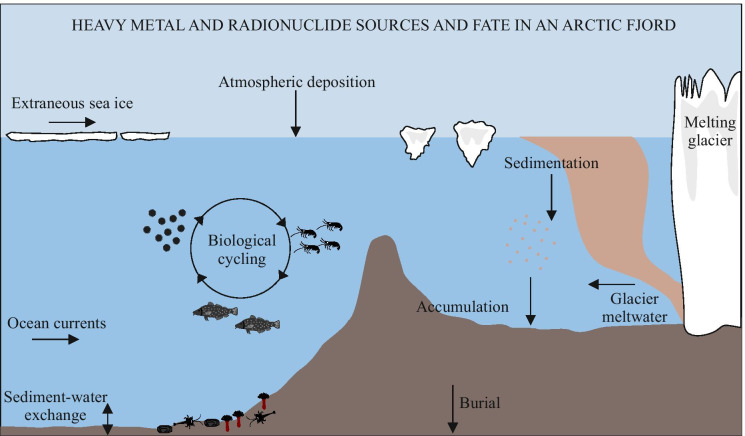
Sources and fate of heavy metals and radionuclides in an Arctic fjord hosting a tidewater glacier (based on Macdonald & Loseto, 2010, and Periáñez et al., 2019)

The distribution of inorganic pollutants is influenced by transport from remote and local sources but is also modified by local conditions in the Arctic environment (Zaborska et al., 2017). These local conditions include seasonality of river flow, melting glacier discharge, intensity of permafrost thawing, precipitation, oceanic circulation, sediment re-suspension rates, composition of suspended matter/sediments and/or the presence of living organisms (Paquin et al., 2003; Stern et al., 2012). Once in the marine environment, the fate of heavy metals and radionuclides depends on their form (dissolved or sorbed on particles). During sedimentation, pollutants can be taken up by marine organisms and cycle in the trophic chain (Fig. 2). Heavy metal and radionuclide accumulation have been found to occur in many Arctic marine organisms (Braune et al., 2005; Campbell et al., 2005; Heldal et al., 2013; Rissanen et al., 1997). Moreover, some metals, e.g. Hg and radionuclides like ^137^Cs, have been found to biomagnify in the marine environment (Dietz et al., 2013; Saremi et al., 2018), and thus monitoring of environmental pollutant levels is necessary (AMAP 2016). Contaminants that accumulate at the sea bottom can be available for the benthic community and be reintroducted to the water column–a process which is enhanced by bioturbation and physical sediment mixing (Thibodaux and Bierman, 2003) or be buried and thus excluded from the environmental cycle (Fig. 2).

### Heavy metals

Heavy metals in the marine environment can be divided into dissolved and undissolved forms. Dissolved metals are present in the water column and can be transported away from the source by water currents. Due to their high affinity to organic matter and fine mineral particles (Ab Razak et al., 1996), heavy metals adsorb on SPM surfaces.

The forms of such heavy metals depend mainly on the chemical characteristics of the metal and certain environmental properties (pH, redox, oxygen content, salinity). When environmental conditions change the form of the heavy metal can also change (AMAP, 2005). This is very visible in the case of metals deposited in marine sediments that are subjected to changes of pH and redox conditions (Solomons & Forstner, 1984). The pH, redox conditions and oxygen content affect multiple processes, such as metal sorption and desorption, dissolution and binding of carbon-bound metals, formation and degradation of soluble and insoluble complex organometallic compounds, formation and dissolution of hydroxides and oxides, and metal precipitation by Fe/Mn oxides (especially in aerobic environments at neutral pH), among many others (Ansari et al., 2004). Oxygen is usually present only in the topmost sediment layers and contaminants deposited in deeper layers many years ago are immobile. However, sediment mixing processes caused by animals (bioturbation) or physical processes (e.g. bottom currents) allow oxygen to enter to the lower sediment layers. Sediment mixing may facilitate re-mobilization of contaminants and their re-introduction to sediment pore waters and subsequently the water column (Thibodeaux & Bierman, 2003) and may induce changes of chemical form from less bioavailable to more. Thus, bottom sediments that have been a sink for contaminants for the last century may now become sources of them (Vintró et al., 2002).

Most studies related to this matter indicate that the toxicity of inorganic contaminants increases with decreasing salinity (Hall & Anderson, 1995; Ytreberg et al., 2011). Heavy metals (e.g. Cd, Hg, Zn) are more bioavailable at lower salinity due to the presence of free metal ions. Increasing salinity decreases the dissolved concentrations of metal ions due to the precipitation of those ions. Thus, in consequence, in more saline waters, the toxicity of metals to organisms is reduced (Park et al., 2014). Ocean waters, due to their higher salinity, have a lower concentration of trace metals, compared to freshwater (Boyle et al., 1974; Hall & Anderson, 1995; Ytreberg et al., 2011). A detailed review of the fate of Hg in the Arctic environment was published by Stern et al. (2012). They indicated several processes that may raise the overall net methylation rate of Hg. These include, for example, extending warmer (ice free) periods and increased intensity of introducing carbon and nutrients and sulphates into the marine ecosystem. Moreover, they indicate that increased input of inorganic Hg from secondary sources (e.g. permafrost thawing, erosion, riverine runoff) would provide more of the mercury substrate required for methylation. On the other hand, an increased photo-demethylation rate and increased MeHg-DOM (dissolved organic matter) binding may reduce the level of MeHg or absorption through the food web.

### Artificial radionuclides

Radionuclides can be divided into non-conservative–primarily adsorbed by solid particles (e.g. plutonium)–and conservative (strontium, caesium, technetium)–which remain dissolved in water and can be transported by water currents (Periáñez et al., 2019). Some radionuclides are also particle-reactive and readily adsorb on SPM and, in consequence, undergo sedimentation to the sea bed.

Changes in radionuclide activity in the marine environment and the prevailing conditions are almost the same as those presented for heavy metals. Progressive climate changes, including an increase in air and water temperature, cause a decrease in the pH and redox value. Along with decreasing pH, there is increased absorption of carbon dioxide by water and then the production of carbonic acid, causing water acidification. Decreasing the redox value of water causes the formation of the so-called dead zones, which are places with reduced oxygen content (AMAP, 2017). Decreasing pH and redox conditions also strongly influence the chemical forms of radionuclides (e.g. Pu, Cs). Their different forms—oxidation states—distinctly influence their affinity to SPM. For instance, in a reducing environment (low pH, low redox, low oxygen), plutonium exists in oxidation state IV or V (Choppin & Morgenstern, 2001; Mitchell et al., 1991). Reduced plutonium is highly particle reactive and is readily scavenged from the water column to sediments. In an oxidizing environment, Pu exists in the form of PuO_2_^+^ and is very soluble in the water column. In this form, it may be transported for long distances and its elimination to marine sediments is very limited.

## Concentration of inorganic contaminants in the Svalbard environment—the current state of knowledge

### Heavy metals

Information on trace metal concentrations in the Arctic environment has been gathered by the Arctic Monitoring and Assessment Program since the 1990s (AMAP, 1998). In addition, and at the same time, the Norwegian national monitoring program started measurements of metal concentrations in atmospheric air in Svalbard (Ny-Ålesund) at the Zeppelin station (NILU database). Classification of heavy metal concentrations in sediments, fauna and flora is possible thanks to the Environmental Quality standards (EQS) established by the Norwegian Environmental Protection Agency (M-608,2016; Veileder 02:^2018^). Other documents enabling the determination of the state of the environment are Environmental Quality Standards given in the EU Water Frame Directive (Lepper, 2005) and Predicted No Effect Concentrations (PNEC) from Risk Assessment Reports available from the EU programme for risk assessment of existing chemicals. A general outline of natural concentrations of heavy metals for rocks of various origins for Svalbard is presented in the literature (Tab. S1). The chemical composition of Svalbard’s overbank deposits was mapped by Ottesen et al. (2010). Natural concentrations of heavy metals in the Hornsund parent material were investigated by Samecka-Cymerman et al. (2011). The heavy metal content of coal deposits was investigated by Headley et al. (1996) for Brøggerhalvøya (West Spitsbergen) and by Orheim et al. (2007) for the Longyear and Svea seam. Lu et al. (2012) established trace metal baseline values for surface sediments in Kongsfjorden.

The metal concentration in the Spitsbergen air varies seasonally (Tab. S2). The Norwegian Institute for Air Research (NILU) has monitored air quality since 1989 (Zeppelin station). The latest research containing an analysis of the measured values of heavy metal concentrations in the air at the Zeppelin station is presented in a report by Bohlin-Nizzetto et al. (2019). They showed a slight decrease in the concentration of As, Pb and Hg and an increase in the concentration of Cu in recent years, compared to 1994. Earlier, e.g. Berg et al. (2004) reported monitoring results of metal concentrations in air at the Zeppelin station from 1994–2002. Only Ni, a metal which is not very toxic, showed a significant downward trend over that time (from 0.19 to 0.07 ng m^−3^), while no significant changes were noticed for other heavy metals. Similar research was conducted later by Conca et al. (2019) in Ny-Ålesund, on the elemental composition of particulate matter PM10. They also indicated the seasonality of the elements’ concentrations, an increase during the spring indicating long-distance transport processes (Arctic haze) and an increase in the summer during periods of increased maritime traffic in the fjords. Based on the isotopic composition of Pb, Bazzano et al. (2015, 2017) indicated that, in spring, at the time of the highest Pb air concentration, long-range transport from Eastern Eurasia is the main Pb source, while in the summer Pb transport from Northern America prevails.

**Fig. 3:**
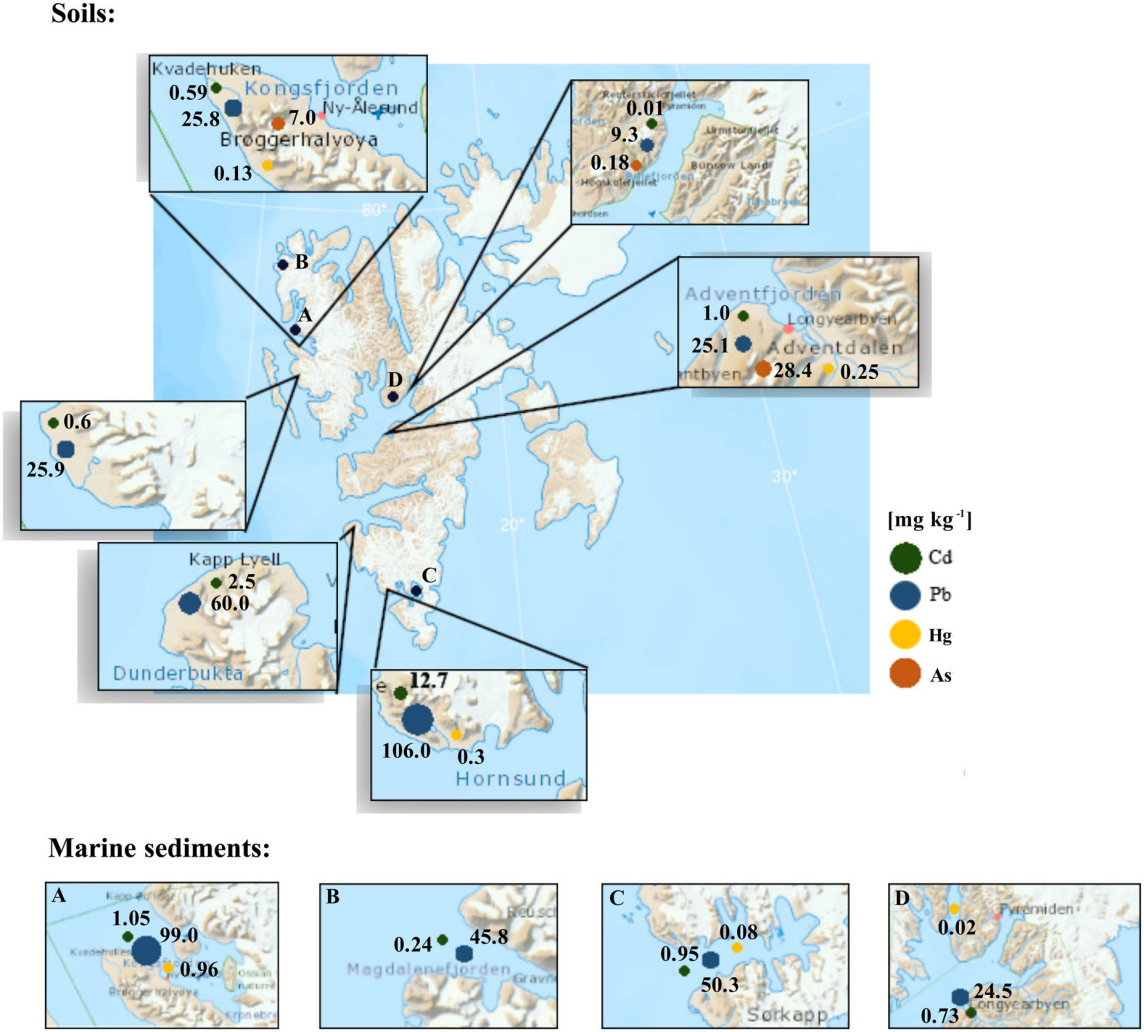
Maximum concentrations of selected heavy metals in soils and marine sediments of Svalbard (References: Table S3)

Elevated heavy metal concentrations have been measured in Spitsbergen soils, e.g. by Negoiţǎ and Ropotǎ (2000), Gulińska et al. (2003), Melke (2006), Chmiel et al. (2009), Wojtuń et al. (2013), Halbach et al. (2017), Ziółek et al. (2017), Aslam et al. (2019) and Łokas et al. (2019) (Fig. 3, Tab. S3). High levels of heavy metal pollution in soil were found by Negoiţǎ and Ropotǎ (2000). Cd, Pb and Cu concentrations were much higher than in other Arctic regions (Greenland and North Siberia). Low concentrations of heavy metals were recorded by Gulińska et al. (2003). The values presented in the article are much lower than for the natural background (Ottensen et al., 2010). However, later studies by Chmiel et al. (2009) and Halbach et al. (2017) showed evident heavy metal contamination in the Scott Glacier Region, Advendalen and Ny-Ålesund. The highest heavy metal concentrations were measured in organically rich soils. The highest concentrations of Cd, Pb, Zn, Cu (Ziółek et al., 2017) and Hg (Wojtuń et al., 2013) were observed in Wedel Jarlsberg Land, and the highest concentrations of As were in Adventdalen (Aslam et al., 2019). In addition, there was a noticeable increase in the concentration of heavy metals in the studies conducted by Ziółek et al. (2017), compared to the earlier studies by Wojtuń et al. (2013), in the Wedel Jarlsberg Land region. For comparison, the latest study by Perryman et al. (2020) measured heavy metal pollution in Alaskan soils and obtained much higher values, exceeding the natural background in this region and ranging from 2.0–720 mg kg^−1^ (mean: 21.1 ± 41.1 mg kg^−1^) for Pb, 0.39–14,900 mg kg^−1^ (mean: 188 ± 1120 mg kg^−1^) for As and 0.01–6090 mg kg^−1^ (mean: 30.4 ± 0.06 mg kg^−1^) for Hg.

Headley (1996) measured increased values of heavy metals (Pb, Zn and Cu) in Kongsfjorden peat. Later, historical Pb enrichment of the peat profile was analysed by Liu et al. (2012). There is a noticeable reduction in Pb concentration compared to earlier studies by Headley (1996) and in the studied core in 1960–2000. Although the measured Pb concentrations were not particularly elevated, a clear anthropogenic signal was found based on the isotopic composition of Pb. Studies performed on peat collected close to Ny-Ålesund have shown that Pb concentrations peaked between 1960 and the 1970s and originated from long-range transport from Western Europe and Russia (Liu et al., 2012). As the peat is well above the surrounding mineral soils, the chemical composition of the surface layers in ombrotrophic peatlands depends on atmospheric deposition. Moreover, the peat surface layers are hydrologically isolated from any influence of local waters (surface and groundwater). Therefore, the peat profile can serve as archival stores of the composition of the atmosphere and can thus provide detailed information on the changing rate of metal deposition from the atmosphere (Souter & Watmough, 2016).

Recently, glacier ice, particularly cryoconites, which are vertical structures on the surface of glaciers, was found to be highly polluted with heavy metals (Singh et al., 2012). The latest studies conducted by Łokas et al., (2016, 2019) indicated an elevated content of heavy metals in these structures (Tab. S3).

**Fig. 4:**
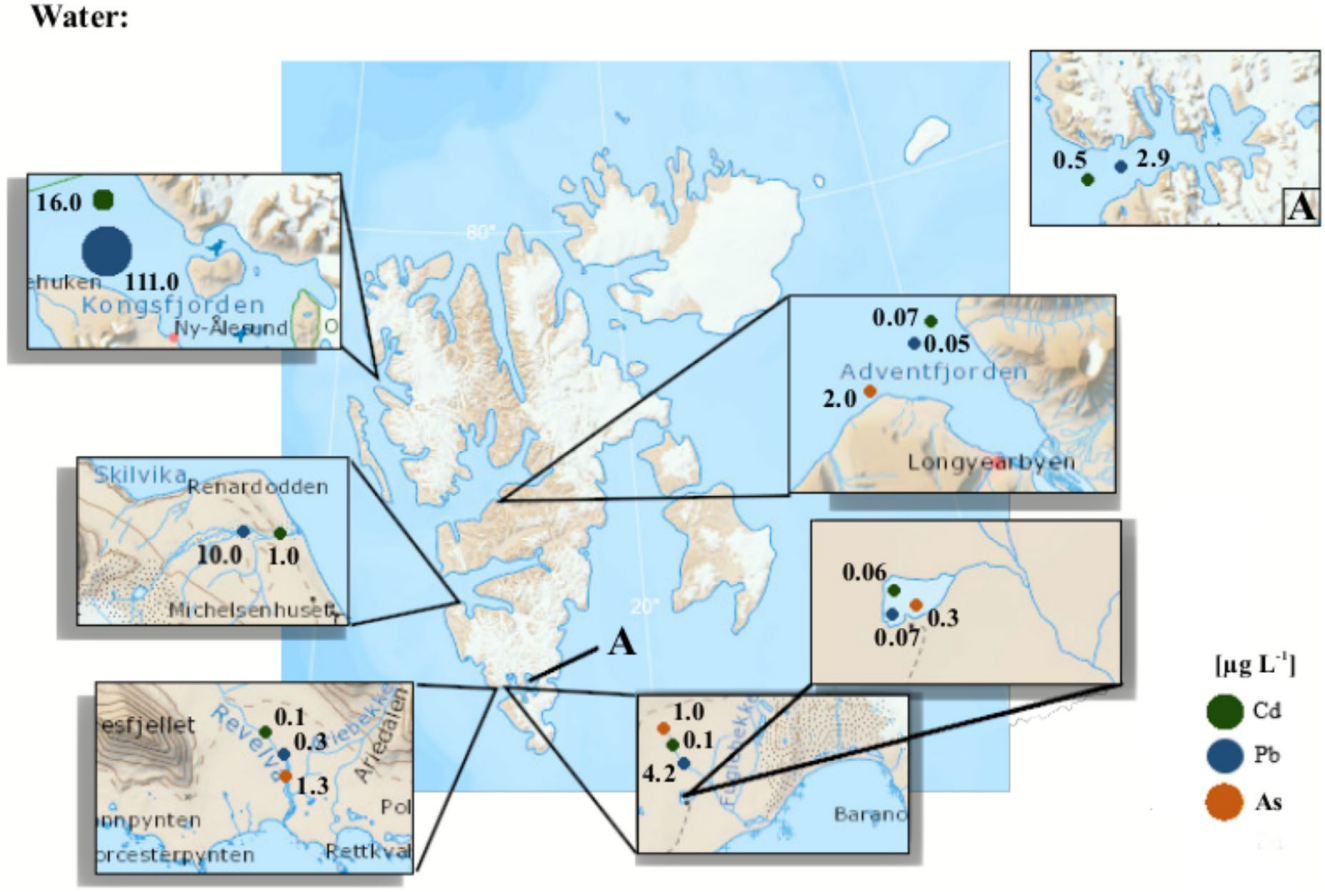
Maximum concentrations of selected heavy metals in surface waters and seawater of Svalbard fjords (References: Table S4)

Research on heavy metals has also been carried out in terrestrial surface water (Fig. 4; Tab. S4). Drbal et al. (1992) were one of the first teams to measure the low concentration of heavy metals in SPM from lake water (Linnévatnet) and spring water from a coastal terrace (Petuniabukta). Significantly higher concentrations of Pb and Zn were recorded in SPM from the Scott River in later studies by Chmiel et al. (2009). Low concentrations of heavy metals have also been recorded in lake water (Chmiel et al., 2009; Kozak et al., 2015), glacier water in the Scott Glacier region (Chmiel et al., 2009) and rainfall (except Zn; Chmiel et al., 2009; Kozak et al., 2015). There was a high concentration of Zn (up to 1378 μg L^−1^) delivered with rainfall in the Fuglebekken catchment. Low concentrations, not exceeding the natural background, were also recorded in riverine water from the Scott Glacier Region (Chmiel et al., 2009), the Fuglebekken catchment (Kozak et al., 2015) and the Revelva river (Kozak et al., 2016). The highest concentrations of Cd, As and Zn were recorded in the Revelva River, while the highest concentrations of Pb and Cu were in main stream water in the Fuglebekken catchment. Measurements were carried out in the Fuglebekken drainage basin to determine the likelihood that atmospheric precipitation is a supplier of Pb, Zn and Cu to surface waters. The concentration of heavy metals in precipitation arriving from long-distance transport and/or secondary sources, e.g. sea salts, was determined by Kozak et al. (2015).

Heavy metals in lake sediments were studied by Sun et al. (2006). Although metal concentrations were not particularly elevated, temporal concentration differences were clearly noticed in sediment cores (Fig. 3; Tab. S3). The highest heavy metal fluxes were observed in the 1980s and 1990s, and a decrease was noted in the twenty-first century. In contrast, Liu et al. (2012) indicated a current overall increase in Pb concentrations in lake sediments. This was attributed to climate-sensitive processes (e.g. increased precipitation and meltwater runoff) caused by the warming of the Arctic. Jiang et al. (2011) reported slightly elevated Hg concentrations, compared to the natural background, in surface lake sediments near Ny-Ålesund. Similar concentrations were measured in other lakes in Spitsbergen: Ossian Sarsfjellet, Ytertjørna, Vassauga and Daltjørna (Drevnick et al., 2012). Gopikrishna et al. (2020) recently analysed sediments collected from three Svalbard lakes for Hg and MeHg content. The mean concentrations were comparable to the values in previous studies for other lakes in the Arctic region. That report pointed out that the Hg in lake sediments was susceptible to MeHg formation. It was noticed that the process of methylation in lake sediments intensified, as a result of increased primary production caused by temperature increase. Consequently, this high productivity could promote Hg accumulation in lakes, accelerating the rate of Hg removal from the water column and subsequent accumulation in bottom sediments (Hudelson et al., 2020; Stern et al., 2012). St Pierre et al. (2018) indicated that glacial rivers are the most important source of Hg for Lake Hazen (Nunavut, Canada). Moreover, permafrost thawing and erosion significantly increased Hg concentrations before entering Chandler Fjord waters. That group also identified the potential for increased Hg concentrations in Arctic ecosystems under the influence of glaciers, as the climate warmed. Klaminder et al. (2010), who researched lead pollution in lakes in the northern part of Sweden, also suggested that the rate of pollution from soil to lakes could increase with climate change.

Typical Pb concentrations in the seawater of Svalbard, at the end of the twentieth century, ranged from 0.1 to 1 μg L^−1^, while at the Russian Arctic coast and sites near mines in Greenland, these levels even reach > 5 μg L^−1^ (Crane et al., 2001). Seawater Cd concentrations in the Svalbard area oscillated around 0.05–0.1 μg L^−1^ but higher Cd concentrations (up to 1 μg L^−1^) have been reported for the Russian Arctic coast and Greenland mining sites (Crane et al., 2001). Currently, research on the content of heavy metals in Spitsbergen (Svalbard) seawater has been studied, e.g. by Bazzano et al. (2014), who investigated heavy metal concentrations in the marine water of Kongsfjorden. They found enrichment of metals in the fjord mouth, introduced along with Atlantic waters, particularly at the end of summer (with the highest value inside the fjord, near the Kongsbreen glacier) (Fig. 4; Tab. S3). Research by Kalinowska et al. (2020) focused on the impact of wastewater on water quality in Spitsbergen. They indicated a small impact on heavy metal pollution from urban activities in Longyearbyen town. Cd concentrations were elevated during the summer (0.07 μg L^−1^), which was linked, inter alia, with discharge from the Adventelva river. Zaborska et al. (2020) studied the temporal and spatial variability of the distribution of heavy metals in the Hornsund fjord in Spitsbergen. This research showed that seawater is contaminated with heavy metals (the Cd concentration reached 303 ng L^−1^ in the particulate fraction and 488 ng L^−1^ in the dissolved fraction), especially in the summer months. Moreover, it was found that the distribution of heavy metals during the year was modified, inter alia, by meltwater discharges from glaciers, atmospheric deposition and oceanic currents, depending on the season.

Heavy metal concentrations in sea ice (Tab. S4) were elevated along with the Greenland current and Fram Strait (Tovar-Sánchez et al. 2010). The highest concentrations of Zn and Cu were observed in the north of Spitsbergen between the parallels of 80°N and 81°N. The latest research by Zaborska et al. (2020) indicated much lower concentrations of these metals in sea ice samples collected in Hornsund.

Several publications have also dealt with trace metal contamination of Spitsbergen fjord marine sediments (Fig. 3; Tab. S3)*.* Most studies conducted so far indicate that the concentration of heavy metals in marine sediments in Svalbard exceeds the natural background values. One of the first studies at the beginning of the twentieth century was carried out by Siegel et al. (2000) in Isfjorden and the Western Spitsbergen shelf. The metal concentrations in the shelf sediments were considered to represent natural concentrations. Elevated concentrations in Isfjorden relative to the shelf suggested an initial problem of contamination from anthropogenic activities. Later, Grotti et al. (2013), Lu et al. (2012) and Frankowski & Frankowska (2014) found elevated metal concentrations in outer Kongsfjorden due to the inflow of Atlantic waters. Bełdowski et al. (2015) studied the concentration of Hg in bottom sediments of fjords of the western coast of Spitsbergen and in bottom sediments of the Barents Sea. The obtained Hg concentrations are in the range of levels identified in the central Arctic Ocean (0.001–0.12 mg kg^−1^), in the Beaufort shelf (0.001–0.13 mg kg^−1^) and along the Greenland coast (0.004–0.28 mg kg^−1^) (Kirk et al., 2012). A study conducted by Zaborska et al. (2017) indicated clear metal enrichment in some Svalbard fjords (Kongsfjorden, Adventfjorden, Hornsund, Rijpfjorden, Smeerenburgfjorden and Magdalenefjorden). Moreover, based on Pb isotopic composition, it was found that up to 85% of Pb originates from anthropogenic sources, mainly in Russia and Europe but also (particularly in the northern fjords) from North America (Zaborska et al., 2017). Mohan et al. (2018) showed an upward trend in the concentration of metals in cores collected in the inner part of Kongsfjorden and observed a high deposition rate since the 1970s, pointing to the impact of industrial emissions. Choudhary et al. (2020) studied the sediments of the Krossfjorden-Kongsfjorden fjord system to determine the environmental toxicity of metals, as well as their origin, mobility and bioavailability. They identified differences in metal concentrations at sites influenced by both glacial activity and Atlantic water masses. However, rock weathering has been identified as the main source of heavy metals for fjords. A study by Kim et al. (2020) compared the content of total Hg in surface sediments in three fjords of Spitsbergen: Hornsund, Dicksonfjorden and Wijdefjorden. The total concentration of Hg associated with organic matter in surface sediments was at its highest in Hornsund, which was attributed to high organic carbon accumulation and intense glacier melting. Recent research by Sagar et al. (2021) indicates significantly increased Cd, Pb, Zn and Cu concentrations in the outer part of Kongsfjorden, compared to previous research in the region.

Other research has focused on heavy metal concentrations in Svalbard fauna and flora (Tab. S5). Godzik et al. (1991) indicated that the concentration of heavy metals within the moss colony on Hornsund’s coast was 1.5–2 times higher than outside the fjord. They believed seabird colonies to be the source of contamination. Much higher Zn and Cu concentrations in moss were found in later studies (Samecka-Cymerman et al., 2011) in West Spitsbergen (Wedel Jarlsberg Land). Elevated concentrations of some heavy metals (Cd and As) were also found in the gonad and intestine of sea urchins, and Laminarian kelps in a later study by Ahn et al. (2009) in Kongsfjorden. Moreover, Jæger et al. (2009) reported low total Hg and MeHg levels in Kongsfjorden zooplankton (muscle and livers), polar cod (muscle and livers), seabird (muscle and livers), Capelin (muscle) and herring (muscle). These studies were also confirmed by Ruus et al. (2015), who obtained equally low concentrations of total Hg and MeHg for Kittiwake, Little auk, Polar cod, Capelin and plankton in Kongsfjorden. Øverjordet et al., (2015a, 2015b) conducted studies on the heavy metal concentrations in the liver and muscles of the black-legged kittiwake, obtaining significantly higher concentration values compared to other species of animals and plants tested in the Kongsfjorden area. They indicated seasonal variability in the diet of organisms as well as migration and moulting as the reason. In the same year, Øverjordet et al., (2015a, 2015b) published studies on Hg and Cd concentrations in Kittiwake and Little Auk while confirming heavy metal contamination of bird in Kongsfjorden and Liedefjorden. The latest research by Pacyna-Kuchta et al. (2020) on little auks in Hornsund also confirmed heavy metal contamination of these organisms, especially in adult birds. Węgrzyn et al. (2016) studied changes in heavy metal concentrations in lichens in the Kaffiøyra Plain (Oskar II Land, NW Spitsbergen) in a transect from the glacier front to the shoreline. They identified the main environmental factors–distance from the shoreline, substrate type and soil pH–which influence heavy metal deposition in lichens. The Cd content in the lichens was almost equal to that measured in the soil (*C. delisei*: 0.10–0.29 mg kg^−1^; soil: 0.12–0.30 mg kg^−1^). More recently, Kłos et al. (2017) also measured the concentration of selected heavy metals in lichen samples. They found high Ni concentrations in samples taken near Longyearbyen, compared to other areas of Spitsbergen, which was attributed to the release of nickel into the atmosphere during coal mining operations in the Longyearbyen area. Węgrzyn et al. (2018) determined the content of selected heavy metals (e.g. Cd, Cu, Fe, Pb and Zn) in reindeer manure. Their main goal was to determine the seasonal variability in heavy metal content in faeces and to compare these values with the nutritional preferences of reindeer throughout the year. They showed lower concentrations of heavy metals in reindeer faeces compared to the concentrations measured in the vegetation they feed on.

### Artificial radionuclides

**Fig. 5:**
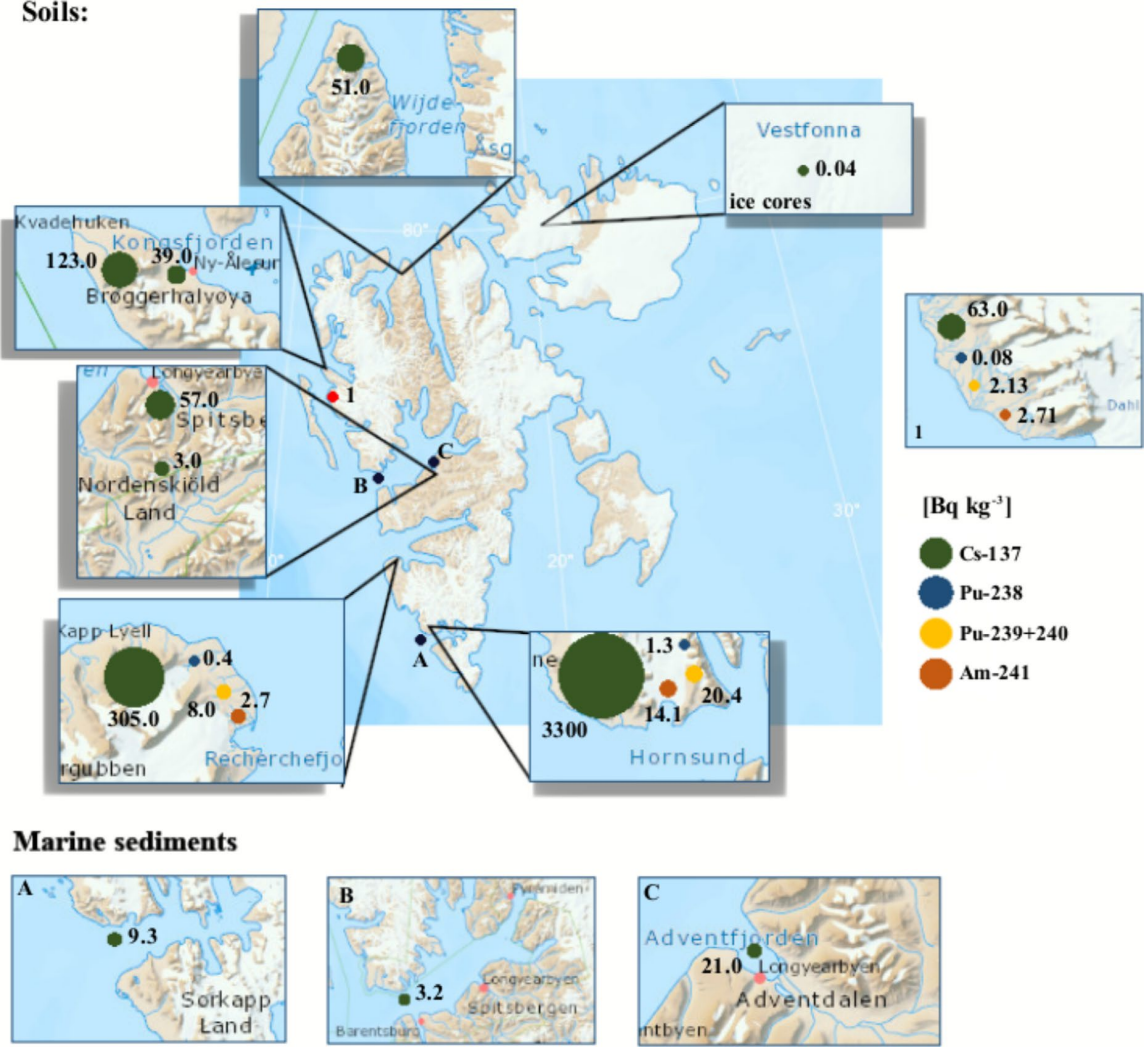
Maximum activity concentrations of selected radionuclides in soils and marine sediments of Svalbard (References: Table S6)

**Fig. 6:**
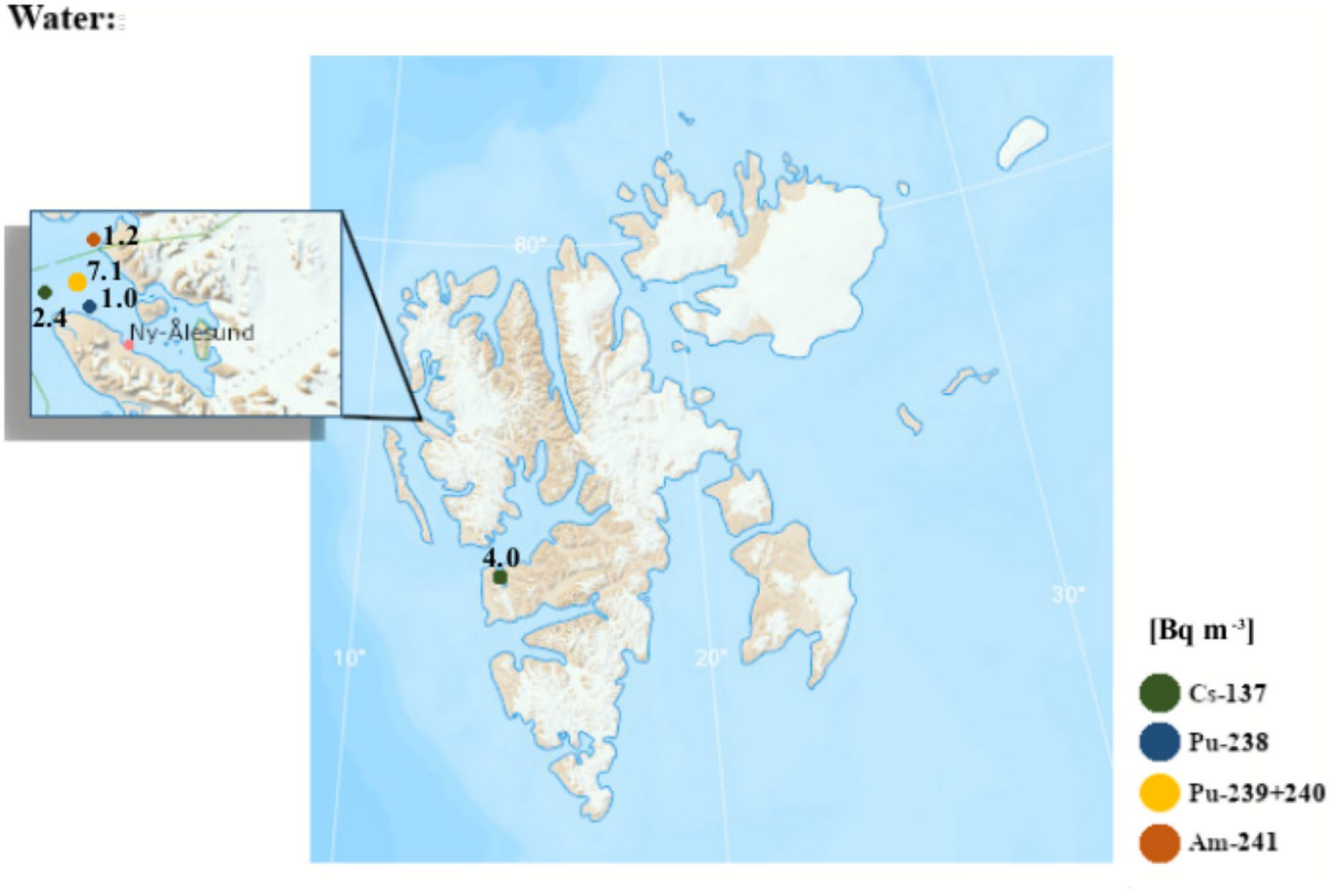
Maximum activity concentrations of selected radionuclides in seawater in different Svalbard regions (References: Table S7)

A literature review recently conducted by Ayoub & Song (2020) includes studies conducted in non-Arctic and Arctic regions which include reference to high precipitation rates and high atmospheric deposition of radionuclides in the Arctic. Moreover, the Arctic was identified as a region with higher radionuclide concentrations compared to other regions (e.g. Antarctica). Figures 5 and 6 present the concentration activities of different artificial radionuclides in various elements of the Spitsbergen environment. The concentration of radionuclides in soils was tested by Dowdall et al., (2003, 2004), Gwynn et al. (2004), Chmiel et al. (2009), Łokas et al., (2013, 2017, 2019) and Kłos et al. (2017). The highest concentrations of ^137^Cs, ^238^Pu, ^239 + 240^Pu and ^241^Am were recorded in Werenskioldbreen (Fig. 5, Tab. S6). Dowdall et al. (2003) indicated that bird droppings could be the main source enriching the soil with radionuclides in Kongsfjorden. These studies were continued and also showed soil contamination with radionuclides from coal mining at Svalbard (Dowdall et al., 2004). The authors found enrichment in natural radionuclide levels caused by anthropogenic activity in the form of coal mining processes (e.g. ^238^U). Research conducted by Łokas et al., (2013, 2019) was carried out in tundra and proglacial zones of mountain glaciers on Svalbard. The activity concentration results showed a very wide range from almost undetectable to extremely high, which was linked to melting of highly enriched cryoconites. Soils of proglacial zones of the Werenskiold Glacier were also found to contain extremely elevated radionuclide activity concentrations, due to melting of glacier ice containing cryoconites (Łokas et al., 2017). Studies on soil radionuclide activity concentrations were conducted by Kłos et al. (2017) near Longyearbyen (Spitsbergen) and indicated that sea aerosols may be an important ^137^Cs source.

Pinglot et al. (1999) examined ^137^Cs activity concentrations in the glacier ice of Svalbard (Kongsvegen, Snøfjella, Vestfonna, Austfonna, Lomonosovfonna and Asgardfonna) (Tab. S6). Radionuclide contamination was found mainly in the reference layers from the Chernobyl nuclear plant explosion and nuclear tests in the 1960s. Łokas et al. (2016) indicated increased activity of radionuclides in cryoconites on the surface of the Hans Glacier in Hornsund. These studies were continued in the cryoconites of the Werenskioldbreen Glacier by Łokas et al. (2017), where activity concentrations for ^137^Cs were much higher than in Hans Glacier. Recent research by Łokas et al. (2019) in the Kaffiøyra region shows activity concentrations of ^137^Cs, ^238^Pu, ^239 + 240^Pu and ^241^Am which indicate higher radionuclide contamination of cryoconite compared to Hans Glacier. Moreover, Cota et al. (2006) and Cámara-Mor et al. (2010) reported extremely high radionuclide activity concentrations in sea ice sediments from different sites of the Arctic Ocean (Resolute Bay, the Nansen Basin and the Fram Strait). The activity of ^137^Cs reached 4 × 10^3^ Bq kg^−1^ (the Nansen Basin) while ^239 + 240^Pu activity concentrations reached 42 Bq kg^−1^ (Resolute Bay).

Appleby (2004) examined ^137^Cs levels in lake sediments from eight lakes located on the west coast of Svalbard, as part of a project dedicated to the investigation of atmospheric pollution and environmental changes in the Arctic (Fig. 5; Tab. S6). He found out that the total activity concentration of deposited ^137^Cs was very variable and ranged from 715 Bq m^−2^ (Arresjøen) to 5032 Bq m^−2^ (Daltjørna).

Gerland et al. (2002) measured ^99^Tc, ^137^Cs, ^238^Pu, ^239 + 240^Pu and ^241^Am levels in marine water, sea ice and seaweed collected in Kongsfjorden and in Greenland and Barents seawater. Gwynn et al. (2004) studied radionuclide contamination of seawater (Fig. 6, Tab. S7). They showed a decrease in ^137^Cs activity concentration to a level which was a factor of 10 lower than that found in the 1980s, which indicates a reduction in discharge of this radionuclide. Based on radionuclide activity ratios, it was found that global fallout from the atmosphere is the dominant radionuclide source. Radionuclide concentrations were measured in different marine environment components (seawater, sediments and biota) of West Spitsbergen by Leppänen et al. (2013). Generally, low ^137^Cs and ^90^Sr activity concentrations are reported for Western Spitsbergen shelf water (Leppänen et al., 2013). In 2020, the latest report was published indicating the concentration of artificial radionuclides in Norway and Svalbard. It presents the results of research on radionuclide concentrations in seawater. The results for ^137^Cs (Ny-Ålesund) presented in the report were lower than in previous studies (Skjerdal et al., 2020).

Research conducted by Heldal et al. (2002) showed quite low pollution of marine sediments with radionuclides (Tab. S6). However, Zaborska (2017) showed an increased flux of ^137^Cs, indicating the great influence of melting glaciers on the phenomenon of fjord pollution. The load of the artificial radionuclide ^137^Cs in the vicinity of glacier outflow was 14 times higher than in the central part of the fjord. ^137^Cs activity concentration was measured to validate sediment dating results by Pawłowska et al. (2017) and earlier by Zajączkowski et al. (2004) in Hornsund and Adventfjorden, and the measured ^137^Cs activity concentrations were low (0.1–7 Bq kg^−1^).

Radionuclides in marine and terrestrial mammals in Svalbard were presented in a report performed by Gwynn et al. (2005) (e.g. in muscles of ringed seals ^137^Cs ranged from 0.4–0.6 Bq kg^−1^, while the corresponding value was 0.3–2.7 Bq kg^−1^ in the muscles of Svalbard reindeers). These figures indicate generally low anthropogenic radioactive contamination in fauna and flora. Benthic echinoderms from Isfjorden and Magdalenefjorden were recently studied by Saniewski and Borszcz (2017) (Tab. S8). This study showed increased concentrations of radionuclides in the bodies of benthic organisms. The authors also point to the serious problem of radionuclide bioaccumulation. However, a recent study by Mezaki et al. (2019) on ^134^Cs and ^137^Cs in animals and plants showed a lack of radioactivity in most of the samples tested. The low activity of ^137^Cs was demonstrated for some lichens and mushrooms.

## Summary and knowledge gaps

Heavy metals and radionuclides are undoubtedly important pollutants of the environment that can affect the ecosystems of Svalbard and the Arctic. It is therefore important to constantly monitor their environmental concentrations. Although the release of pollutants from secondary sources has recently been described in the literature, no regular and comprehensive study has been performed. Global climate change, melting glaciers, thawing permafrost or increased riverine runoff can lead to the release of pollutants previously deposited on surfaces and increase the load of inorganic pollutants in the marine environment. Significant scientific advances have been made regarding the effects of climate change on the fate of inorganic pollutants in the Arctic, and these advances show increased discharge of metals (e.g. mercury from melting permafrost and glaciers). Although there are some empirical data to support the conceptual predictions presented by Macdonald et al. in 2005, for example Stern et al. (2012), Mohan et al. (2018) and St Pierre et al. (2019), there is still no complete picture of the environmental fate of inorganic contaminants, especially radionuclides, in Svalbard. Contamination of the Arctic environment by organic substances, particularly new emerging pollutants (e.g. pharmaceutical residues) should also be thoroughly studied.

## Supplementary Information

Below is the link to the electronic supplementary material.

Supplementary file1 (DOCX 16 KB)

Supplementary file2 (DOCX 15 KB)

Supplementary file3 (DOCX 19 KB)

Supplementary file4 (DOCX 23 KB)

Supplementary file5 (DOCX 20 KB)

Supplementary file6 (DOCX 15 KB)

Supplementary file7 (DOCX 16 KB)

Supplementary file8 (DOCX 16 KB)
