# Supplementary material for: Sources, fate and distribution of inorganic contaminants in the Svalbard area, representative of a typical Arctic critical environment–a review
Source: Environ Monit Assess. 2021 Oct 14;193(11):724. doi: 10.1007/s10661-021-09305-6 (PMC8516776; doi:10.1007/s10661-021-09305-6)
Supplement: Supplementary file 1 — Supplementary file1 (DOCX 16 KB) [file 10661_2021_9305_MOESM1_ESM.docx]

**Table S1.** Natural concentrations of heavy metals for rocks of various origins for Svalbard

| **Localization** | **Samples collected** | **Heavy metal** | **Concentration**  **[mg kg^-1^]** | **Reference** |
| --- | --- | --- | --- | --- |
| **Overbank deposits** | | | | |
| Spitsbergen  (Svalbard) | 1986 | Cd | 0.01-3.8  0.15* | Ottesen et al., 2010 |
|  |  | Pb | 0.5-84.8  12.0* |  |
|  |  | As | 0.5-108.0  6.8* |  |
|  |  | Hg | 0.01-0.25  0.03* |  |
|  |  | Zn | 11.0-169.0  68.0* |  |
|  |  | Cu | 0.92-1.63  20.0* |  |
| **Parent rock** | | | | |
| Hornsund  (Spitsbergen) | 2008 | Cd | 0.01-0.14  0.04* | Samecka-Cymerman et al., 2011 |
|  |  | Pb | 1.9-42.0  11.0* |  |
|  |  | Zn | 17.0-170.0  58.0* |  |
|  |  | Cu | 1.2-25.0  11.0* |  |
| **Coal deposits** | | | | |
| Ny-Ålesund coal | 1993 | Pb | 4.8-5.2  5.0* | Headley, 1996 |
|  |  | Zn | 16.0-17.0  16.6* |  |
|  |  | Cu | 3.2-3.4  3.32* |  |
| Longyear seam | 1978-2003 | Cd | 1.0-2.0 | Orheim et al., 2007 |
|  |  | Pb | 1.0-13.0  2.0* |  |
|  |  | As | 1.0-31.0 |  |
| Svea seam |  | Cd | 1.0 |  |
|  |  | Pb | 2.0-6.0  3.0* |  |
|  |  | As | 1.0-4.0  2.0* |  |
| **Marine sediments** | | | | |
| Kongsfjorden  (Spitsbergen) | 2009 | Cd | 0.14 | Lu et al., 2013 |
|  |  | Pb | 17.46 |  |
|  |  | Hg | 0.001 |  |
|  |  | Zn | 70.49 |  |
|  |  | Cu | 25.14 |  |

*median
