# Supplementary material for: Sources, fate and distribution of inorganic contaminants in the Svalbard area, representative of a typical Arctic critical environment–a review
Source: Environ Monit Assess. 2021 Oct 14;193(11):724. doi: 10.1007/s10661-021-09305-6 (PMC8516776; doi:10.1007/s10661-021-09305-6)
Supplement: Supplementary file 2 — Supplementary file2 (DOCX 15 KB) [file 10661_2021_9305_MOESM2_ESM.docx]

**Table S2.** Literature data on the concentration of heavy metals [ng m^-3^] in air on Spitsbergen

| **Localization** | **Samples collected** | **Heavy metal** | **Concentration**  **[ng m^-3^]** | **Reference** |
| --- | --- | --- | --- | --- |
| **Air** | | | | |
| Zeppelin | 1994-2002 | Cd | 0.01-0.03 | Berg et al., 2004 |
|  |  | Pb | 0.48-0.83 |  |
|  |  | As | 0.05-0.7 |  |
|  |  | Hg (g)* | 1.50-1.79 |  |
|  |  | Zn | 1.2-1.9 |  |
|  |  | Cu | 0.25-0.41 |  |
| Zeppelin | 2019 | Cd | 0.01-0.05 | Bohlin-Nizzetto et al., 2019 |
|  |  | Pb | 0.02-0.77 |  |
|  |  | As | 0.01-0.13 |  |
|  |  | Hg (g)* | 0.92-1.66 |  |
|  |  | Zn | 0.4-6.9 |  |
|  |  | Cu | 0.08-1.15 |  |
| Ny-Ålesund** | 2010-2013 | Cd | 0.00-0.13 | Conca et al., 2019 |
|  |  | Pb | 0.00-1.22 |  |
|  |  | As | 0.00-0.24 |  |
|  |  | Zn | 0.00-18.00 |  |
|  |  | Cu | 0.00-0.61 |  |

*mercury in gas phase

**measurements for particulate matter PM10
