# Supplementary material for: Sources, fate and distribution of inorganic contaminants in the Svalbard area, representative of a typical Arctic critical environment–a review
Source: Environ Monit Assess. 2021 Oct 14;193(11):724. doi: 10.1007/s10661-021-09305-6 (PMC8516776; doi:10.1007/s10661-021-09305-6)
Supplement: Supplementary file 3 — Supplementary file3 (DOCX 19 KB) [file 10661_2021_9305_MOESM3_ESM.docx]

**Table S4.** Literature data on the concentration [µg L^-1^] of dissolved heavy metals in precipitation (rain), glacier meltwater and riverine and marine water on Spitsbergen

| **Localization** | **Samples collected** | **Heavy metal** | **Concentration**  **[****µg L^-1^]** | **Reference** |
| --- | --- | --- | --- | --- |
| **Lake water** | | | | |
| Scott Glacier Region | 2005 | Cd | <1 | Chmiel et al., 2009 |
|  |  | Pb | <1-10 |  |
|  |  | Zn | <1-10 |  |
|  |  | Cu | <1 |  |
| Fuglebekken catchment | August 2010 and October 2010 | Cd | 0.06 | Kozak et al., 2015 |
|  |  | Pb | 0.01-0.07 |  |
|  |  | As | 0.1-0.3 |  |
|  |  | Zn | 0.5-2.1 |  |
|  |  | Cu | 0.1-0.9 |  |
| **Rainfall** | | | | |
| Scott Glacier Region | 2005 | Cd | <1.0 | Chmiel et al., 2009 |
|  |  | Pb | <1.0-10 |  |
|  |  | Zn | 5-50 |  |
|  |  | Cu | <1 |  |
| Fuglebekken catchment | August 2010 and September 2012 | Cd | 0.08-1.08 | Kozak et al., 2015 |
|  |  | Pb | 0.01-3.77 |  |
|  |  | As | 0.1-0.4 |  |
|  |  | Zn | 0.50-1378 |  |
|  |  | Cu | 0.1-5.0 |  |
| **Glacier’s water** | | | | |
| Scott Glacier Region | 2005 | Cd | <1 | Chmiel et al., 2009 |
|  |  | Pb | <1-10 |  |
|  |  | Zn | 1-10 |  |
|  |  | Cu | <1 |  |
| **Riverine water** | | | | |
| Scott Glacier Region | 2005 | Cd | <1 | Chmiel et al., 2009 |
|  |  | Pb | <1-10 |  |
|  |  | Zn | <1-10 |  |
|  |  | Cu | <1 |  |
| Fuglebekken catchment | August 2010 and September 2012 | Cd | 0.06-0.12 | Kozak et al., 2015 |
|  |  | Pb | 0.01-4.20 |  |
|  |  | As | 0.1-1.0 |  |
|  |  | Zn | 0.5-9.9 |  |
|  |  | Cu | 0.1-2.6 |  |
| Revelva river | 2010-2012 | Cd | 0.05-0.14 | Kozak et al., 2016 |
|  |  | Pb | 0.01-0.3 |  |
|  |  | As | 0.1-1.3 |  |
|  |  | Zn | 0.5-14.2 |  |
|  |  | Cu | 0.0-0.1 |  |
| **Sea water** | | | | |
| Kongsfjorden | 2012 | Cd | 0.001-0.02 | Bazzano et al., 2014 |
|  |  | Pb | 0.009-0.11 |  |
|  |  | Zn | 0.02-0.46 |  |
|  |  | Cu | 0.01-0.26 |  |
| Adventfjorden-Longyearbyen System | summer and autumn 2018 | Cd | 0.01-0.07 | Kalinowska et al., 2020 |
|  |  | Pb | 0.01-0.05 |  |
|  |  | As | 1.0-2.0 |  |
|  |  | Zn | 0.07-2.14 |  |
|  |  | Cu | 0.1-1.07 |  |
| Hornsund | from April to September 2016 | Cd | 0.002-0.5 | Zaborska et al., 2020 |
|  |  | Pb | 0.02-2.91 |  |
|  |  | Zn | 3.97-33.30 |  |
|  |  | Cu | 0.14-6.28 |  |
| **Sea ice** | | | | |
| Fram Strait | 2007 | Zn | 46.99-606.30 | Tovar‐Sánchez et al., 2010 |
|  |  | Cu | 5.31-30.54 |  |
| Hornsund | from April to September 2016 | Cd | 0.001-0.24 | Zaborska et al. 2020 |
|  |  | Pb | 0.001-0.13 |  |
|  |  | Zn | 4.74-37.02 |  |
|  |  | Cu | 0.06-2.32 |  |
