# Supplementary material for: Sources, fate and distribution of inorganic contaminants in the Svalbard area, representative of a typical Arctic critical environment–a review
Source: Environ Monit Assess. 2021 Oct 14;193(11):724. doi: 10.1007/s10661-021-09305-6 (PMC8516776; doi:10.1007/s10661-021-09305-6)
Supplement: Supplementary file 4 — Supplementary file4 (DOCX 23 KB) [file 10661_2021_9305_MOESM4_ESM.docx]

**Table S5.** Literature data on the concentration of heavy metals [ng m^-3^] in flora and fauna on Spitsbergen

| **Localization** | **Samples collected** | **Species** | **Heavy metal** | **Concentration**  **[mg kg^-1^]** | **Reference** |
| --- | --- | --- | --- | --- | --- |
| Hornsund | 1986 | Moss  (Sanionia uncinata) | Cd | 0.81-6.33 | Godzik, 1991 |
|  |  |  | Pb | 4.3-13.7 |  |
|  |  |  | Zn | 12.3-46.9 |  |
|  |  |  | Cu | 1.2-10.0 |  |
| Kongsfjorden | 2003-2004 | sea urchins-gonad | Cd | 0.97 | Ahn et al., 2009 |
|  |  |  | Pb | 0.05 |  |
|  |  |  | As | 12.4 |  |
|  |  |  | Zn | 62.7 |  |
|  |  |  | Cu | 2.03 |  |
|  |  | sea urchins-intestine | Cd | 3.21 |  |
|  |  |  | Pb | 0.31 |  |
|  |  |  | As | 16.0 |  |
|  |  |  | Zn | 54.9 |  |
|  |  |  | Cu | 2.79 |  |
|  |  | Laminarian kelps | Cd | 2.05 |  |
|  |  |  | Pb | 0.08 |  |
|  |  |  | As | 62.7 |  |
|  |  |  | Zn | 8.20 |  |
|  |  |  | Cu | 1.13 |  |
| Kongsfjorden | 2006 | Zooplankton - muscle | TotHg | n.d.* | Jæger et al., 2009 |
|  |  | Zooplankton - liver | MeHg | n.d. |  |
|  |  | Capelin - muscle | TotHg | n.d. |  |
|  |  | Herring - muscle | TotHg | n.d.-0.02 |  |
|  |  | Polar cod - muscle | TotHg | n.d.-0.02 |  |
|  |  |  | MeHg | 0.004-0.02 |  |
|  |  | Seabirds - muscle | TotHg | 0.01-0.66 |  |
|  |  | Polar cod - liver | TotHg | n.d.-0.01 |  |
|  |  | Seabirds - liver | TotHg | 0.02-2.00 |  |
|  |  |  | MeHg | 0.023-1.6 |  |
| Wedel Jarlsberg Land | 2008 | Sanionia uncinata | Cd | 0.41-0.82 | Samecka-Cymerman et al., 2011 |
|  |  |  | Pb | <LOD**-10.98 |  |
|  |  |  | Zn | 78-254 |  |
|  |  |  | Cu | 2.1-15 |  |
| Kongsfjorden | 2007 | Kittiwake | TotHg | 0.0-0.01 | Ruus et al., 2015 |
|  |  |  | MeHg | 0.0-0.01 |  |
|  |  | Little auk | TotHg | 0.0002-0.0005 |  |
|  |  |  | MeHg | 0.0002-0.0004 |  |
|  |  | Polar cod | MeHg | 0.00 |  |
|  |  | Capelin | MeHg | 0.00 |  |
|  |  | Plankton | TotHg | 0.00 |  |
|  |  |  | MeHg | 0.00 |  |
| Kongsfjorden | 2007 | black-legged kittiwake - liver | Cd | 15.0-60.0 | Øverjordet et al., 2015 |
|  |  |  | Hg | 0.5-5.0 |  |
|  |  |  | Zn | 90.0-145.0 |  |
|  |  |  | Cu | 20.0-25.0 |  |
|  |  | black-legged kittiwake - muscle | Cd | 0.0-5.0 |  |
|  |  |  | Hg | 0.0-1.0 |  |
|  |  |  | Zn | 40.0-50.0 |  |
|  |  |  | Cu | 15.0-18.0 |  |
| Kongsfjorden | 2008-209 | Kittiwake | Hg | 1.9-2.5 | Øverjordet et al., 2015 |
|  |  |  | Cd | 25.0-48.0 |  |
|  |  | Little auk | Hg | 1.0 |  |
|  |  |  | Cd | 15.0-20.0 |  |
| Liedefjorden |  | Kittiwake | Hg | 1.9-2.5 |  |
|  |  |  | Cd | 30.0-40.0 |  |
|  |  | Little auk | Hg | 0.8 |  |
|  |  |  | Cd | 10.0-18.0 |  |
| Kaffiøyra Plain (Oskar II Land, NW Spitsbergen) | 2012 | Lichen  (Cetrariella delisei) | Cd | 0.10-0.29 | Węgrzyn et al., 2016 |
|  |  |  | Pb | 7.9-18.1 |  |
|  |  |  | Zn | 29-45 |  |
|  |  |  | Cu | 2.2-7.4 |  |
| Bolterdalen | 2015 | Reindeer faeces | Cd | 0.001-0.008 | Węgrzyn et al., 2018 |
|  |  |  | Pb | 0.06-0.84 |  |
|  |  |  | Zn | 15.0-38.0 |  |
|  |  |  | Cu | 0.02-0.21 |  |
| Hornsund | 2016-2017 | Adult body | Cd | <LOD-2.15 | Pacyna-Kuchta et al., 2020 |
|  |  |  | Pb | <LOD-10.98 |  |
|  |  |  | As | <LOD-14.43 |  |
|  |  |  | Hg | 0.58-2.36 |  |
|  |  |  | Zn | 78-254 |  |
|  |  |  | Cu | 8.81-58.35 |  |
|  |  | Adult throat | Cd | <LOD |  |
|  |  |  | Pb | <LOD-20.22 |  |
|  |  |  | As | <LOD |  |
|  |  |  | Hg | 1.45-17.20 |  |
|  |  |  | Zn | 62-696 |  |
|  |  |  | Cu | 2.56-67.97 |  |
|  |  | Chick body feather | Cd | <LOD |  |
|  |  |  | Pb | <LOD |  |
|  |  |  | As | <LOD-11.50 |  |
|  |  |  | Hg | 0.25-0.96 |  |
|  |  |  | Zn | 70-132 |  |
|  |  |  | Cu | 19.07-42.23 |  |
|  |  | Chick down | Cd | 0.18-1.82 |  |
|  |  |  | Pb | <LOD-1.24 |  |
|  |  |  | As | 0.10-3.38 |  |
|  |  |  | Hg | 0.64-2.03 |  |
|  |  |  | Zn | 38.25-97.66 |  |
|  |  |  | Cu | 5.99-17.35 |  |
|  |  | Eggshells | Cd | <LOD |  |
|  |  |  | Pb | <LOD |  |
|  |  |  | As | 0.02-12.00 |  |
|  |  |  | Hg | <LOD |  |
|  |  |  | Zn | 0.60-3.88 |  |
|  |  |  | Cu | 0.09-1.07 |  |

* not detected

**Limit of Detection
