# Supplementary material for: Sources, fate and distribution of inorganic contaminants in the Svalbard area, representative of a typical Arctic critical environment–a review
Source: Environ Monit Assess. 2021 Oct 14;193(11):724. doi: 10.1007/s10661-021-09305-6 (PMC8516776; doi:10.1007/s10661-021-09305-6)
Supplement: Supplementary file 5 — Supplementary file5 (DOCX 20 KB) [file 10661_2021_9305_MOESM5_ESM.docx]

**Table S6.** Literature data on the activity concentration of radionuclides [Bq kg^-1^] in particulate fraction of selected terrestrial and marine environment compounds (surface soils, suspended matter, sediments, cryconites) on Spitsbergen

| **Localization** | **Samples collected** | **Radionuclide** | **Activity concentration**  **[Bq kg^-1^]** | **Reference** |
| --- | --- | --- | --- | --- |
| **Surface soils** | | | | |
| Kongsfjorden | 2000, 2002 | ^137^Cs | 0.3 – 123.0 | Dowdall et al. 2003 |
| around Ny-  Alesund. | 2001 | ^137^Cs | 0.3 – 39.0 | Dowdall et al. 2004 |
| Northern Spitsbergen | 2001 | ^137^Cs | 31.0 – 51.0 | Gwynn et al. 2004 |
| Southern Spitsbergen |  | ^137^Cs | <0.7 – 3.0 |  |
| Scott Glacier  Region, Spitsbergen | 2005 | ^137^Cs | 0.55 – 13.31 | Chmiel et al. 2009 |
| south-western part of  the Wedel Jarlsberg Land (Svalbard) | 2007 | ^137^Cs | <0.1 - 305 | Łokas et al. 2013 |
|  |  | ^238^Pu | <0.01 – 0.40 |  |
|  |  | ^239+240^Pu | <0.01 – 7.96 |  |
|  |  | ^241^Am | <0.02 – 2.71 |  |
| Werenskioldbreen | 2005 and 2007 | ^137^Cs | 2 – 3300 | Łokas et al., 2017 |
|  |  | ^238^Pu | 0.03 – 1.33 |  |
|  |  | ^239+240^Pu | 0.05 – 20.4 |  |
|  |  | ^241^Am | 0.05 – 14.1 |  |
| Longyearbyen | 2014 | ^137^Cs | 12.7 – 57.0 | Kłos et al., 2017 |
| Kaffiøyra region | 2014 | ^137^Cs | <2 – 63 | Łokas et al. 2019 |
|  |  | ^238^Pu | <0.02 – 0.08 |  |
|  |  | ^239+240^Pu | <0.02 – 2.13 |  |
|  |  | ^241^Am | <0.03 – 0.90 |  |
| **Suspended matter (SPM)** | | | | |
| Scott Glacier  Region – Scott river | 2005 | ^137^Cs | 0.12 | Chmiel et al. 2009 |
| **Cryconites and ice cores** | | | | |
| Vestfonna (North Svalbard) – ice cores | between 1981 - 1998 | ^137^Cs | 0.0 – 0.04 | Pinglot et al., 1999 |
| Hans Glacier, Hornsund | 2011 | ^137^Cs | 89 - 678 | Łokas et al. 2016 |
|  |  | ^238^Pu | 0.07 – 1.27 |  |
|  |  | ^239+240^Pu | 1.65 – 16.62 |  |
|  |  | ^90^Sr | 15 - 101 |  |
| Werenskioldbreen Glacier | 2005 | ^137^Cs | 700-4500 | Łokas et al., 2017 |
| Kaffiøyra region | 2014 | ^137^Cs | <3 - 2030 | Łokas et al. 2019 |
|  |  | ^238^Pu | <0.02 – 2.10 |  |
|  |  | ^239+240^Pu | <0.02 – 42.77 |  |
|  |  | ^241^Am | <0.20 – 24.48 |  |
| **Lake sediments** | | | | |
| Ossian Sarfjellet | 1993 and 1995 | ^137^Cs | 701 | Appleby 2004 |
| Yterjorna |  | ^137^Cs | 320 |  |
| Vassauga |  | ^137^Cs | 293 |  |
| Daltjorna |  | ^137^Cs | 507 |  |
| Tenndammen |  | ^137^Cs | 83 |  |
| Arresjoen |  | ^137^Cs | 1861 |  |
| Birgervatnet |  | ^137^Cs | 928 |  |
| **Marine sediments** | | | | |
| Spitsbergen–Bear Island area | 1998-1999 | ^137^Cs | 0.7 – 6.3 | Heldal et al., 2002 |
|  |  | ^239+240^Pu | 0.98 – 2.37 |  |
|  |  | ^238^Pu | 0.033 – 0.096 |  |
|  |  | ^241^Am | 0.37 – 0.85 |  |
| West of Spitsbergen |  | ^137^Cs | 3.9 – 9.3 |  |
| Adventfjorden | 2001-2002 | ^137^Cs | 1.0 – 21.0 | Zajączkowski et al., 20014 |
| West of Spitsbergen | 2008-2009 | ^137^Cs | 0.9 – 6.2 | Leppänen et al. 2013 |
|  |  | ^90^Sr | 0.1 – 4.2 |  |
| Hornsund | 2012 | ^137^Cs | 0.1 – 8.5 | Pawłowska et al. 2017 |
| Hornsund | 2016 | ^137^Cs | <0.1 – 7.7 | Zaborska, 2017 |
