# Supplementary material for: Sources, fate and distribution of inorganic contaminants in the Svalbard area, representative of a typical Arctic critical environment–a review
Source: Environ Monit Assess. 2021 Oct 14;193(11):724. doi: 10.1007/s10661-021-09305-6 (PMC8516776; doi:10.1007/s10661-021-09305-6)
Supplement: Supplementary file 6 — Supplementary file6 (DOCX 15 KB) [file 10661_2021_9305_MOESM6_ESM.docx]

**Table S7.** Literature data on the concentration of dissolved radionuclides in seawater on Spitsbergen

| **Localization** | **Samples collected** | **Radionuclide** | **Activity concentration [Bq/m^3^]** | **Reference** |
| --- | --- | --- | --- | --- |
| **Seawater** | | | | |
| Kongsfjorden | 2000 | ^137^Cs | 1.84 – 2.32 | Gerland et al., 2002 |
|  |  | ^99^Tc | 0.21 – 0.26 |  |
|  |  | ^239+240^Pu | 0.007 ± 0.001 |  |
|  |  | ^238^Pu | <0.0004 |  |
|  |  | ^241^Am | 0.001 ± 0.001 |  |
| Kongsfjorden | 2001 | ^137^Cs | 2.33 – 2.41 | Gwynn et al., 2004 |
|  |  | ^99^Tc | 0.25 – 0.29 |  |
|  |  | ^239+240^Pu | 6.1 – 7.1 |  |
|  |  | ^238^Pu | 0.7 - 1 |  |
|  |  | ^241^Am | 0.7 – 1.2 |  |
| West of Spitsbergen | 2008-2009 | ^137^Cs | 0.3 – 4.0 | Leppänen et al. 2013 |
|  |  | ^90^Sr | 1.6 – 8.0 |  |
| Ny-Alesund | 2015-2017 | ^137^Cs | 0.9-1.0 | Skjerdal et al., 2020 |
| Greenland Sea,  Western Svalbard | 2015 | ^137^Cs | 1.5 |  |
|  |  | ^239+240^Pu | 4.0 |  |
|  |  | ^241^Am | 2.2 |  |
| **Sea ice** | | | | |
| Kongsfjorden | 2000 | ^137^Cs | 0.40 | Gerland et al., 2002 |
