# Supplementary material for: Sources, fate and distribution of inorganic contaminants in the Svalbard area, representative of a typical Arctic critical environment–a review
Source: Environ Monit Assess. 2021 Oct 14;193(11):724. doi: 10.1007/s10661-021-09305-6 (PMC8516776; doi:10.1007/s10661-021-09305-6)
Supplement: Supplementary file 7 — Supplementary file7 (DOCX 16 KB) [file 10661_2021_9305_MOESM7_ESM.docx]

**Table S8.** Literature data on the concentration of heavy metals [ng m^-3^] in flora and fauna on Spitsbergen

| **Localization** | **Samples collected** | **Species** | **Radionuclide** | **Activity concentration [Bq kg^-1^]** | **Reference** |
| --- | --- | --- | --- | --- | --- |
| Isfjorden | 2009-2012 | Henricia sanguinolenta | ^137^Cs | <0.9-37.3 | Saniewski and Borszcz, 2017 |
|  |  | Ophiopolis oculeata | ^137^Cs | <7.3-11.9 |  |
|  |  | Strongulocentiatus drabalenis | ^137^Cs | <0.9-4.3 |  |
| Magdalenefjorden |  | Strongulocentiatus drabalenis | ^137^Cs | <2.6 |  |
| Isfjorden | 2011-2013 | Bearded seal | ^137^Cs | N.D | Mezaki et al., 2019 |
|  |  | Ringed seal | ^137^Cs | N.D |  |
|  |  | Glaucous gull | ^137^Cs | N.D |  |
|  |  | Svalbard rock ptarmigan | ^137^Cs | N.D |  |
|  |  | Lagopus muta hyperborea | ^137^Cs | N.D |  |
|  |  | Atlantic puffin | ^137^Cs | N.D |  |
|  |  | Northern fulmar | ^137^Cs | N.D |  |
|  |  | Atlantic cod | ^137^Cs | N.D |  |
|  |  | Arctic char | ^137^Cs | N.D |  |
|  |  | Shorthorn sculpin | ^137^Cs | N.D |  |
|  |  | Myoxocephalus scorpius | ^137^Cs | N.D |  |
|  |  | Jellyfish | ^137^Cs | N.D |  |
|  |  | Pink salmon | ^137^Cs | N.D |  |
|  |  | Oncorhynchus gorbuscha | ^137^Cs | N.D |  |
|  |  | Swimming snail | ^137^Cs | N.D |  |
|  |  | Sea gooseberry | ^137^Cs | N.D |  |
|  |  | Lichen | ^137^Cs | N.D-46.4 |  |
|  |  | Mushroom | ^137^Cs | N.D-36.2 |  |
|  |  | Grass | ^137^Cs | N.D-18.3 |  |
|  |  | Seaweed | ^137^Cs | N.D |  |
